# Improving rational thermal comfort prediction by using subpopulation characteristics: A case study at Hermitage Amsterdam

**DOI:** 10.1080/23328940.2017.1301851

**Published:** 2017-03-20

**Authors:** Rick Kramer, Lisje Schellen, Henk Schellen, Boris Kingma

**Affiliations:** a Department of the Built Environment, Eindhoven University of Technology, Eindhoven, The Netherlands; b Department of Human Biology and Movement Sciences, NUTRIM School of Nutrition and Translational Research in Metabolism, Maastricht University Medical Center, Maastricht, The Netherlands; c Avans University of Applied Sciences, Tilburg, The Netherlands; d Department of Energy Technology, Eindhoven University of Technology, Eindhoven, The Netherlands

**Keywords:** calibration, PMV, survey study, thermal comfort

## Abstract

This study aims to improve the prediction accuracy of the rational standard thermal comfort model, known as the Predicted Mean Vote (PMV) model, by (1) calibrating one of its input variables “metabolic rate,” and (2) extending it by explicitly incorporating the variable running mean outdoor temperature (RMOT) that relates to adaptive thermal comfort. The analysis was performed with survey data (*n* = 1121) and climate measurements of the indoor and outdoor environment from a one year-long case study undertaken at Hermitage Amsterdam museum in the Netherlands. The PMVs were calculated for 35 survey days using (1) an *a priori* assumed metabolic rate, (2) a calibrated metabolic rate found by fitting the PMVs to the thermal sensation votes (TSVs) of each respondent using an optimization routine, and (3) extending the PMV model by including the RMOT. The results show that the calibrated metabolic rate is estimated to be 1.5 Met for this case study that was predominantly visited by elderly females. However, significant differences in metabolic rates have been revealed between adults and elderly showing the importance of differentiating between subpopulations. Hence, the standard tabular values, which only differentiate between various activities, may be oversimplified for many cases. Moreover, extending the PMV model with the RMOT substantially improves the thermal sensation prediction, but thermal sensation toward extreme cool and warm sensations remains partly underestimated.

## Introduction

A museum is a public space visited by subpopulations that vary widely in characteristics such as age, gender, socio-cultural and socio-economic background, and also thermal expectations. The indoor climate of museums is primarily important for collection preservation. However, a museum also aims to provide a thermally comfortable indoor environment for its visitors.

Current guidelines and specifications for the design and operation of the indoor environment of buildings are formulated in standards such as ASHRAE Standard 55 and EN-ISO 7730.^1,2^ These standards provide a rational methodology to assess the indoor thermal environment of buildings. The standards contain two distinct thermal comfort models. One of the models reflects a heat balance model that predicts the Predicted Mean Vote (PMV) based on the follo-wing physical characteristics: human metabolic heat production, clothing level, external work, air temperature, mean radiant temperature, relative air humidity, and air speed.^3^ The percentage people dissatisfied (PPD) is calculated by the PMV, and a building is considered to provide sufficient thermal comfort when the PPD remains below 10%, i.e., a PMV between −0.5 and +0.5. The application of the PMV-PPD model typically results in a constant indoor environment with a little variation other than a winter and summer scenario to account for seasonal clothing adjustments. The other model is data driven, involving data from 160 buildings including 21,000 data sets from across the globe and is called the adaptive comfort model.^4^ This model implicitly includes the ability of people to adapt both physiologically and psychologically to climate zones and seasonal changes in outdoor climate.^5^ The major outcome of the adaptive model is that especially for naturally ventilated (NV) buildings, comfortable indoor temperatures are higher when the running mean outdoor temperature (RMOT) is higher and vice versa.^5,6^ Although the PMV model does allow taking into account behavioral adaptations (e.g., changes in physical activity or clothing), it does not allow us to include physiologic (e.g., acclimatization) and psychologic adaptations (e.g., expectations). Various studies have shown that hybrid PMV-Adaptive models, thus including seasonal adaptations, and adaptive effects on expectations and the metabolic rate can improve the thermal sensation prediction. For a recent overview, see Refs. 5 and 7.

The physical input variables of the PMV model, e.g., humidity and operative temperature, are, in general, well defined and measured accurately. However, the human metabolic rate and clothing level often lack accuracy, because these are too hard to measure.^8^ For that reason, specialists rely on standard tables providing values for separate garments from which a clothing ensemble can be constructed indicating the mean clothing level (e.g., male business suit ≈0.155 m^2^ K/W ≡ 1 clo). Also, tables are used for metabolic heat production that links the activity type to metabolic rate (e.g., light office work ≈58–70 W/m^2^ ≡ 1–1.2 Met). However, applying a single equivalent metabolic heat production rate for a specific activity to the entire population may be oversimplified with respect to the actual biologic variation.^9^ Variation in human resting metabolic rate is mainly explained by differences in body composition and size.^10^ Average body composition and size differ among subpopulations. For example, females have, in general, less total mass and relatively more fat mass than males, and elderly have increased fat mass relative to the younger adults. These differences contribute to variation in body heat production; however, this is currently not reflected in the standard tables for the metabolic rate.

To provide a more accurate estimation of metabolic rate, empirical equations exist to estimate the basal metabolic rate of a subpopulation based on their characteristics. For example, the Harris and Benedict equation revised by Roza and Shizgal^11^ is used to calculate the basal metabolic rate (*BMR* [W]) according to

(1) $$BMR_{\text{ females}}=\;0.0484\left(13.397W+4.799H-5.677A+88.362\right)$$

(2) $$BMR_{\text{ males}}=\;0.0484\left(9.247W+3.098H-4.330A+447.593\right)$$

in which 0.0484 is the conversion factor from kcal/day to J/s, *W* is the body weight [kg], *H* the body height [cm], and *A* the age [y]. The basal metabolic rate (≈0.8 Met) differs from the resting metabolic rate (≈1 Met) as it is measured in supine position, fasted state, and in a thermoneutral environment. Equation (2) predicts that the heat equivalent of a 1-Met activity level is equal to 58 W/m^2^ for an 81 kg, 1.80 m, 20 y old male (body surface area calculated from the Dubois equation), which is equal to what the ASHRAE standard table notes. However, for a 69 kg, 1.74 m, 20-y old female, Equation (1) predicts 51 W/m^2^ that is considerably lower than her male counterpart. Table 1 presents the subpopulation's average height and weight for Dutch adults and elderly males and females.^12^ Based on equations (1) and (2), the expected resting metabolic rate of older adults is on average 9% lower than that of mid-aged adults.

**Table 1.** Average height and weight for two age categories in the Dutch population.^12^

	Male			Female		
	30–40 [y]	65–75 [y]	Δ [%]	30–40 [y]	65–75 [y]	Δ [%]
Height [cm]	182.5	177.3	−3	168.9	164.9	−2
Weight [kg]	83.2	84.5	2	69.9	71.4	2
Surface area [m^2^]	2.05	2.02	−2	1.8	1.78	−1
Met. rate [W/m^2^]	55.2	50	−9	49.3	45	−9

Note: Body surface area is calculated using the Dubois equation. The metabolic rate is calculated using the Harris and Benedict equation and scaled to 1 Met (original output is basal metabolic rate and assumed at 0.8 Met).

Thus, based on physiologic characteristics, the metabolic rate of older adults is expected to be lower than that of adults. Consequently, we hypothesize that if the metabolic rate is calibrated, by fitting the predicted thermal sensation (PMV-model) to the actual thermal sensation (measurements), a similar difference between age groups will be observed. Moreover, in line with the previous literature on adaptive behavior, the second hypothesis is that predicted thermal sensation can be improved by including the RMOT.

## Methods

The analyses were performed with data from a 1-y-long case study undertaken at Hermitage Amsterdam museum in the Netherlands. The resulting database comprises surveys (*n* = 1121), outdoor climate measurements, and indoor climate measurements. The methodology for constructing the underlying database used in this study has been published before in Ref. 13. Therefore, here only a brief overview is provided.

### Case study: Museum Hermitage Amsterdam

**Figure 1.**
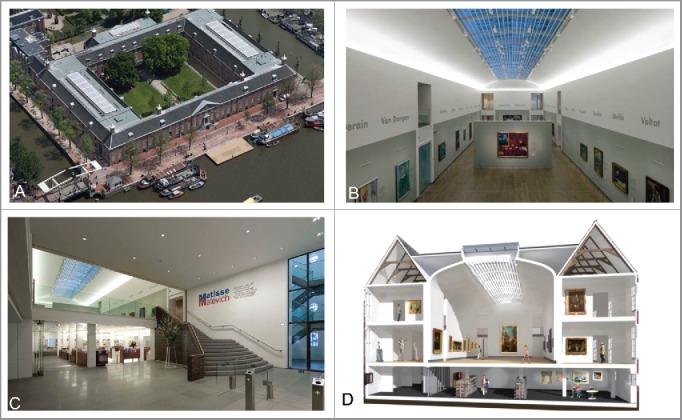
(A) Aerial view of Hermitage Amsterdam museum. (B) One of two main exhibition rooms with a large glass roof. (C) The entrance stairway from the lobby to the main exhibition room with an air curtain to reduce air exchange. (D) A cross section of one side of the building showing the main exhibition room and adjacent cabinets. Figure source: Ref. 14.

Hermitage Amsterdam (Fig. 1) is a sister museum of the State Hermitage museum in St. Petersburg, Russia. The museum is located in Amsterdam, the Netherlands, and is housed in a late 17th-century building. The most recent renovation dates from 2007 to 2009 when the building was transformed into a state-of-the-art museum (see Fig. 1). The historic building envelope was conserved and insulated from the inside. An all-air heating, ventilation, and air-conditioning system was installed to condition the exhibition areas. The indoor climate specifications used under normal operating conditions are 21 ± 0.5 °C and 50 ± 1% RH without seasonal adjustments. For the current study, the indoor temperature has been varied to cover a range of operative temperatures (19.5–24 °C) over the year while maintaining relative humidity at 50%.

Figure 1A shows the layout of the building. The building has a symmetric floor plan: Two identical exhibition wings can be recognized by their glass skylights on the left and right side roofs. This study focuses on “de Keizersvleugel,” the exhibition wing shown on the right side of Figure 1A. The exhibition area consists of the main hall (Fig. 1B) and adjacent cabinets (Fig. 1D). Visitors enter the exhibition area via a stairway from the foyer (Fig. 1C). The ceiling of the main exhibition hall consists in part of a large glass roof with interior sunblinds that are almost permanently closed.

The museum is opened 7 days/week from 10 to 17 h and has welcomed 7,000–11,000 visitors per week depending on the exhibition. Most individuals visited the museum on Sunday, Tuesday, and Wednesday, whereas the least number came on Monday.

### Data acquisition

**Figure 2.**
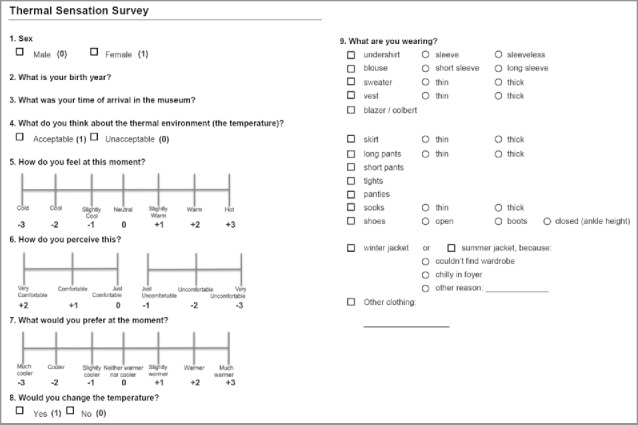
The used survey including numerical transcription used for statistical analysis.

The current study was conducted from February 2015 to December 2015. Data acquisition comprised surveys (subjective), and indoor and outdoor climate measurements (objective). Surveys were conducted on Wednesdays and Thursdays between 11 h and 14 h, because during these time slots most visitors were welcomed. At least 30 surveys were collected each survey day. The survey and measurement location in the museum was always in the cabinets on the second floor in “de Keizersvleugel,” see Figure 1D. This location was best suitable since most museum visitors had spent more than 30 min inside upon reaching this location. Indoor climate measurements on a measurement grid showed that the indoor climate throughout the museum was very stable and homogeneous, verifying that the indoor climate conditions on the location of the surveys were representative for the rest of the museum. Surveys were provided in both Dutch and English. Figure 2 shows the survey including the numerical transcription used for the statistical analysis.

The survey included nine questions concerning gender, age, time in the museum, acceptability of the thermal indoor environment, thermal sensation, thermal comfort, thermal preference, desire to change the temperature, and clothing level. Besides, the time when each survey was conducted was noted. The clothing level was determined based on participants' responses to the survey. The transcription to Clo-value was based on numerical values provided by ASHRAE.^1^

Indoor measurements consisted of air temperature, globe temperature, relative air humidity, and airspeed. Table 2 provides specifications concerning the measurement instruments used. ASHRAE^1^ recommends to perform the measurements at the following heights for standing subjects: 0.1 m, 1.1 m, and 1.7 m. The limited number of sensors available meant that measurements could be taken only at a single height. It was deemed suitable to position the instruments at head/neck level helping to better monitor the thermal conditions at the level of face and neck, which, because of being continually exposed, could be most vulnerable. Therefore, the measurement height was 1.7 m. The sampling interval for the indoor measurements was 1 s. The indoor operative temperature was used for further analysis and calculated as the mean of the air temperature and the radiant temperature. This is considered valid since airspeed remained below 0.2 m/s. Outdoor air temperature and relative air humidity were acquired from the museum's weather station via the building management system. The sampling interval for the outdoor measurements was 16 min.

**Table 2.** Specification of instruments used for indoor climate measurements.

Variable	Range	Accuracy	Sensor
Air temperature [°C]	−80–150	±0.10	NTC type DC95
Radiant temperature [°C]	−55–80	±0.05	NTC U-type
Air relative humidity [%]	0–100	±3.00	Humitter® 50YX
Airspeed [m/s]	0.05–5.00	0.02 ± 1.5%	SensoAnemo 5132SF

### Analysis procedure

**Figure 3.**
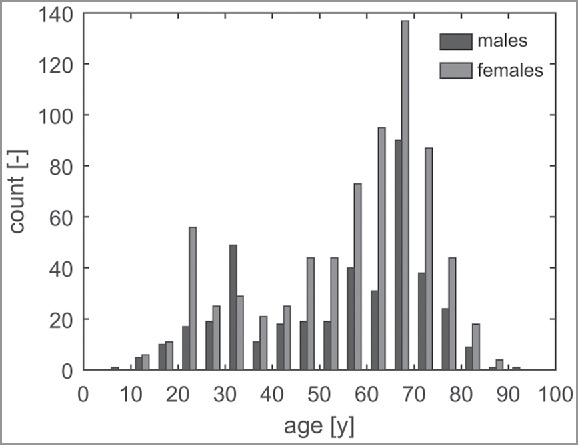
Number of males and females per age group.

*Step 1*: The database comprises 1,248 samples. Samples including erratic values or missing values and samples of respondents who were in the museum for less than 20 min were excluded resulting in a total of 1,121 samples that were used for analysis. Figure 3 shows the number of males and females categorized by age. The museum's visitor population consists predominantly of older females, followed by older males.

As mentioned in Introduction, the metabolic rate of older adults (65–75 y) is expected to be 9% lower than that of younger adults (30–40 y). Table 3 shows the number of respondents in these age categories in the current data set. This subset consists mostly of elderly, particularly older females.

**Table 3.** Number of respondents of the subpopulations used for further analysis.

Age [y]	*N* males [−]	*N* females [−]
30–40	60	50
65–75	128	224

*Step 2*: The standard procedure for PMV has been used as a reference allowing us to compare the prediction performance. The standard PMV was calculated using the input variables as shown in Table 4. The variable “metabolism” is unknown from either objective measurements or subjective survey responses. Thus, for the standard PMV model, a constant metabolic rate (Met) was chosen from tabular values: The heat equivalent of 1 Met is defined as 58.2 W/m^2^.^15^ The standard input value for a museum environment is *a priori* assumed to be 1.5 Met, i.e., the mean of standing relaxed (1.2 Met) and walking around (1.7 Met),^16^ rounded to one decimal place. The prediction accuracy was determined by calculating the PMV for each survey day, including daily mean values for the input parameter Clo and the daily measured environmental variables, and using 1.50 Met for the metabolic rate. The PMVs of the survey days have been compared with the actual mean votes (AMVs), which are the daily means of the TSVs and the general prediction accuracy was assessed using linear regression between actual versus predicted measurements (Actual = *a*_1_ Predicted + *a*_0_). The prediction accuracy is determined by the combination of explained variance (*R*^2^-value) and intercept (*a*_0_) and slope of the regression line (*a*_1_): A perfect prediction accuracy implies 100% explanation of the variance (*R*^2^ = 1), zero bias (*a*_0_ = 0), and a slope equal to the line of identity (*a*_1_ = 1).

**Table 4.** Input variables of the PMV model and methods of determination.

Input parameter	Determination
Metabolism [W/m^2^]	Unknown
External work [W/m^2^]	0
Radiant temperature [°C]	Measured
Air temperature [°C]	Measured
Relative air humidity [−]	Measured
Clothing level [clo]	Transcription from survey responses
Air speed [m/s]	Measured

*Step 3*: The PMV model has been applied to every individual respondent, and the metabolic rate of each individual was calibrated to make an exact fit of the PMV to the individual TSV of that respondent. Hence, in total 1,121 unique values for metabolic rates have been estimated using the local optimization solver “fminsearch” in MATLAB release 2015b.^17^ This method naively assumes that all residuals are caused by metabolic rate. However, individual thermal sensation is determined by many other factors such as adaptation, personal preference, and cultural background.^18^ These other influences are not explicitly defined in the PMV model. Therefore, any variance introduced by other factors than the metabolic rate will be discounted in this procedure and may cause a significant error in the estimation of individual metabolic rates. Given the large sample size, the influence of non-metabolic rate variables is assumed to be equally distributed over the entire population. Nevertheless, the error introduced by other factors may result in extremely low and high metabolic rates. To reduce the influence of extreme values on the analysis, the non-parametric Wilcoxon-rank-sum test was applied to test whether the distribution of estimated metabolic rates differed between the age groups (adults of 30–40 y versus older adults of 65–75 y). Statistical significance was assumed when *p* < 0.05.

*Step 4*: The PMV has been calculated for each survey day using the median metabolic rate, further referred to as PMV_met_. The PMV_mets_ were compared with the AMVs and linear regression was used to assess the prediction performance as explained in *Step 2*.

*Step 5*: The Pearson correlation coefficient *r* was calculated between the residuals (AMV – PMV_met_) and all other variables from the database. Following the recommendation of Kenny for studies involving subjective human responses, only correlations ≥ 0.3 were further analyzed and discussed.^19^ If a relevant correlation was found, linear regression was applied to describe the linear relation between the residuals and the specific variable and the PMV_met_ model was extended using that linear relation.

The reference outdoor temperature RMOT was calculated according to

(3) $$RMOT\;=\frac{T_{\text{e},\text{i}}+0.8T_{\text{e},\text{i}-1}+0.4T_{\text{e},\text{i}-2}+0.2T_{\text{e},\text{i}-3}}{2.4}\;$$

where *T_e,i_* is the average outdoor temperature of the survey day, *T*_e,i-1_ the average of the day before, etc. The average is the arithmetic mean of the minimum and maximum outdoor temperature of the given day. This reference outdoor temperature has been proposed by van der Linden et al.^20^ and is an implementation of the exponentially weighted RMOT by Nicol.^21^

## Results

### Indoor climate conditions

**Figure 4.**
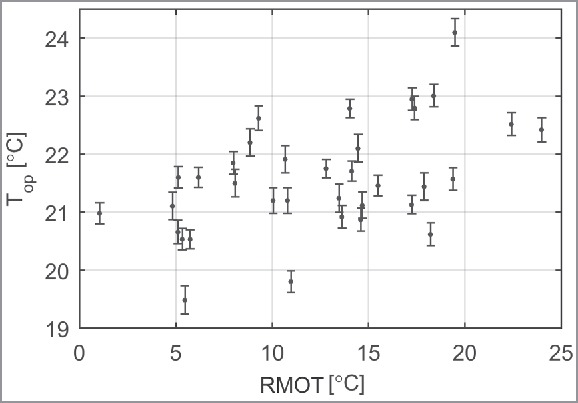
Mean operative temperatures ± SD during the survey days as a function of the running mean outdoor temperature (RMOT) according to Equation (3).

The indoor temperature was varied over the year to include a range of indoor operative temperatures, while maintaining relative air humidity at 50%. Figure 4 shows the operative temperatures during the survey days as a function of the RMOT. The indoor temperatures range from 19.5 °C to 24 °C. The small standard deviations indicate that temperature was accurately controlled.

### Step 1: Standard value for metabolic rate

**Figure 5.**
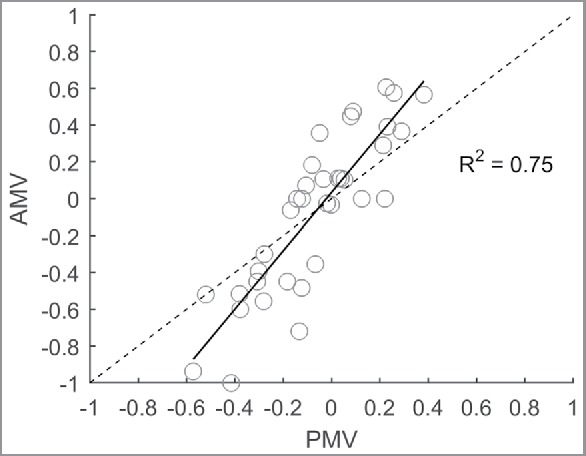
AMV versus PMV using an assumed metabolic rate of 1.5 Met and the line of identity (dashed).

Figure 5 shows the prediction accuracy of the PMV model using a standard input of 1.50 Met and using the daily means for the rest of the measured input parameters. The prediction performance of the PMV model is described by the following linear regression: AMV = 1.584 PMV – 0.034, *R*^2^ = 0.75, *p* < 0.01. The PMV model provides a good explanation of the variance; however, the slope differs significantly from the line of identity (95% confidence interval of *a*_1_: 1.284–1.884) that indicates that there is a clear underestimation of the thermal sensation toward more extreme AMVs (−0.5 < AMV < 0.5).

### Step 2: Calibrating metabolic rates

**Figure 6.**
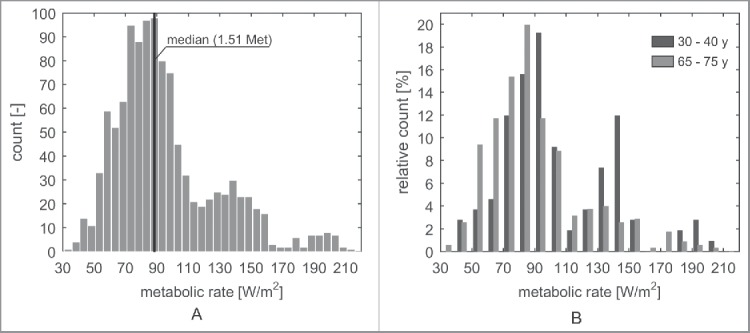
(A) All identified metabolic rates. (B) Identified metabolic rates of adults and elderly. The age groups 30–40 y and 65–75 y were chosen because information on these groups was available from the database of the Dutch central bureau for statistics (CBS) shown in Table 3.

Figure 6A shows the calibrated metabolic rates of the museum visitors found by minimizing the error between the AMVs and the PMVs. The calibration of the metabolic rates was successful for each individual respondent: The remaining prediction errors (ε = TSV − PMV) are all smaller than 1 × 10^−5^. The histogram shows a multimodal distribution for the entire population that is indicative for the existence of subgroups with distinct metabolic rates. Figure 6B shows the fitted metabolic rates of the subpopulations adults (30–40 y) and elderly (65–75 y) showing a shift between both subpopulations' maximum metabolic rates. For the next step, the median value of the distribution is used to represent the majority of the population.

**Figure 7.**
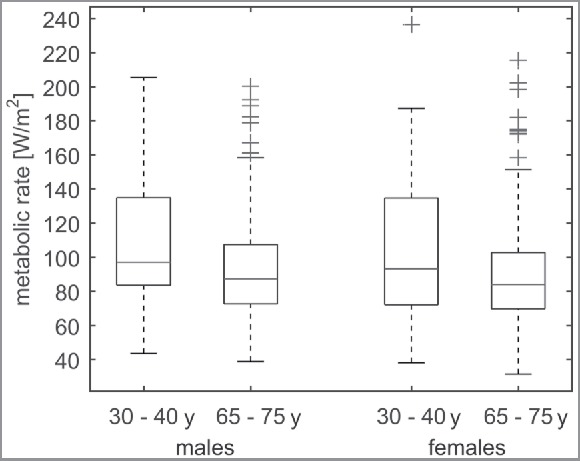
Calibrated metabolic rates of males and females for different age categories. The age groups 30–40 y and 65–75 y were chosen because information on these groups was available from the database of the Dutch central bureau for statistics (CBS) shown in Table 3.

Figure 7 shows the calibrated metabolic rates of four subpopulations categorized with respect to age and gender equal to the introduced subpopulations in Table 1. The median metabolic rate is 10.3% higher in adult males compared with elderly males (97 W/m^2^ vs. 87 W/m^2^, *p* < 0.01) and on average 9.7% higher for adult females compared with elderly females (93 W/m^2^ vs. 84 W/m^2^, *p* < 0.01). The *a priori* estimation of activity level (1.50 Met) appears appropriate for this case study (median is 1.51 Met), especially for the elderly. However, the metabolic rates of the mid-aged adult subpopulations deviate substantially from this *a priori* value (1.67 Met for adult males, 1.50 Met for older males, 1.60 Met for adult females, and 1.44 Met for older females). Moreover, Figure 7 shows that the calibrated metabolic rates vary considerably among the individual respondents.

Then, the median of the calibrated metabolic rates (1.51 Met) was used as input for the PMV model instead of the *a priori* standard value (1.50 Met), and the daily means of the measured variables were used for the rest of the inputs. There is no significant change or improvement, which is to be expected since the *a priori* assumed metabolic rate (1.50 Met) is very close to the actual metabolic rate (1.51 Met).

### Step 3: Extending the PMV model

The prediction errors of the PMV model, i.e., the residuals (AMV − PMV_met_), show relevant correlations with several variables indicating that the unexplained variance may be partly explained by including additional inputs. Table 5 presents the Pearson correlation coefficients and their *p*-values. From all variables in the database, the RMOT, the clothing (Clo), and the indoor air relative humidity (RH_i_) reveal relevant correlations, of which RMOT shows the strongest correlation. The correlations of RMOT and Clo are significant, whereas RH_i_'s correlation is not significant.

**Table 5.** Relevant correlations between prediction residuals and the variables RMOT (running mean outdoor temperature), Clo (clothing insulation), and RH_i_ (indoor air relative humidity).

	RMOT	Clo	RH_i_
Pearson *r*	−0.54	0.38	−0.32
*p*	<0.01	0.03	0.06

The influence of RMOT is included in the extended PMV model, further referred to as PMV*, by applying linear regression to the residuals (AMV − PMV_met_) and RMOT. Hence, PMV* may be calculated according to

(4) $${\text{PMV}}^{*}={\text{PMV}}_{\text{met}}\;-0.024\text{ RMOT}+0.264$$

**Figure 8.**
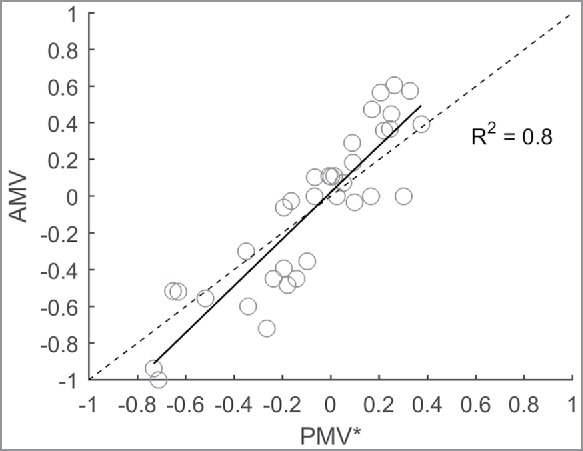
AMV versus PMV* and the line of identity (dashed).

Figure 8 shows the prediction accuracy of the PMV* model. The prediction performance of the PMV* model is described by the following linear regression equation: AMV = 1.272 PMV* + 0.020, *R*^2^ = 0.80, *p* < 0.01. The PMV* model provides a better explanation of the variance and the prediction of the thermal sensation toward more extreme AMVs has improved.

Table 6 shows the correlations between the new residuals (AMV − PMV*) and the aforementioned variables (see Table 5). Since the information of RMOT is included in the PMV* model, the correlation has diminished. Moreover, the correlations between the residuals and the variables Clo and RH_i_ are negligible indicating that the clothing behavior and indoor RH correlate with RMOT. Although RH_i_ was controlled by humidification and dehumidification in the museum, RH_i_'s correlation with RMOT may be explained by seasonal adjustments in the system set point for RH_i_.

**Table 6.** Correlations between prediction residuals and the variables RMOT (running mean outdoor temperature), Clo (clothing insulation), RH_i_ (indoor air relative humidity).

	RMOT	Clo	RH_i_
Pearson *r*	<0.01	−0.08	0.01
*p*-value	1	0.63	0.95

Further analysis shows that the residuals (AMV − PMV*) are not significantly explained by Gender (*r* = −0.19, *p* = 0.08) or any other variable but are strongly correlated with the variable thermal preference (*r* = −0.88, *p* < 0.01). Linear regression has been used to extend the PMV* model with the inclusion of thermal preference votes. The resulting PMV** model is estimated according to

(5) $${\text{PMV}}^{**}={\text{PMV}}^{*}\;-0.742\text{ Preference}+0.035$$

**Figure 9.**
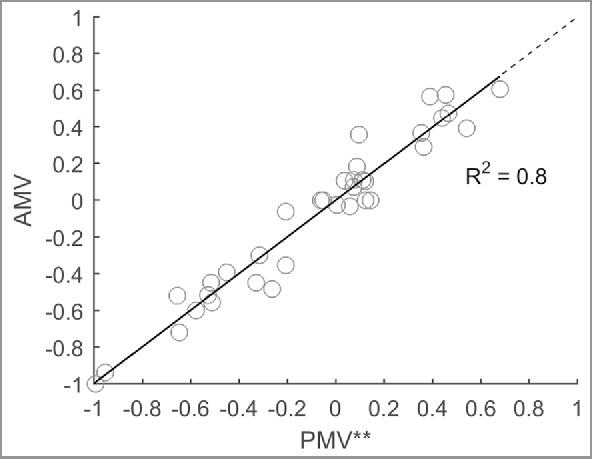
AMV compared with PMV**. Regression analysis shows the relation is very close to the line of identity (dashed): AMV = 0.997 PMV**-0.0002.

The equation indicates a substantial contribution of thermal preference to the thermal sensation prediction. Figure 9 shows the prediction performance of the PMV** model. Inclusion of the thermal preference does explain the underestimation of the PMV* model toward more extreme AMVs: The new slope is nearly identical to the line of identity but does not improve the explained variance (*R*^2^-value remains 0.8).

## Discussion and conclusions

This study presents a case study at Hermitage Amsterdam museum differentiating the metabolic rate for different age groups and accounting for the outdoor temperature (RMOT) to improve the rational prediction of thermal sensation.

The inputs of Fanger's Predicted Mean Vote (PMV) model comprise both environmental variables (temperature, RH_i_, and airspeed) and personal variables that may vary among individuals (metabolic rate and clothing level). Earlier studies have shown that in many cases AMV and PMV do not correlate strongly and that this may be explained by inaccurate values for input parameters such as for metabolic rate.^7^ Furthermore, thermal expectation has also been shown to skew the relation between AMV and PMV in warm environments.^7^ An extensive review of the PMV model identified that improved prediction of thermal comfort can be achieved through improving the validity of the PMV model, better specification of the model's input variables, and accounting for outdoor thermal conditions and special groups.^22^

The metabolic rates of 1121 respondents in a museum case study have been estimated using the PMV model. The variable metabolic rate has been calibrated by minimizing the difference between the respondent's TSV and the predicted thermal sensation vote (PMV), i.e., the residuals. This implies the assumption that the residuals are only caused by the variable metabolic rate. However, individual thermal sensation is determined by many other factors such as adaptation, personal preference, culture, or background difference.^18^ Consequently, the identified metabolic rates (Fig. 6a) cover a wide range including few extreme values, i.e., values lower than 70 W/m^2^ and higher than 150 W/m^2^. These extreme values are likely to be unrealistic. The median has been used for further analysis instead of the mean to minimize the effect of the extreme values. Although the individual metabolic rates need to be interpreted with extreme care, the differences in median metabolic rates between different subpopulations are considered to be meaningful.

The median calibrated metabolic rates of various subpopulations differ significantly: The results show that the metabolic rate of elderly appears to be 10% lower compared with the metabolic rate of adults (*p* < 0.01). This difference is consistent with what could be expected based on physiologic data (see Table 1).^23^ Noteworthy is that the results show a substantial variation among individual respondents (Fig. 7).

The *a priori* assumed metabolic rate for physical activity in a museum environment (1.5 Met) appears to be close to the median of the estimated metabolic rates (1.51 Met). This may be explained by the fact that the visitors' population was dominated by older females (1.44 Met) in this case study as shown in Figure 3. However, given that the metabolic rates appear to differ significantly among subpopulations, the *a priori* derived metabolic rate may be significantly off in other cases. For example, museums visited predominantly by younger people require a different assumption for the metabolic rate (1.6–1.7 Met). Hence, we conclude that the tabular values, e.g., in Ref. 16 may not be appropriate for specific subpopulations in an environment in which humans generally have an elevated activity level compared with an office environment, such as a museum.

Earlier studies show that occupants of air-conditioned office buildings (HVAC-buildings) are barely influenced by the RMOT and that the adaptation process is more prominent in NV office buildings.^4^ However, this study shows that the prediction errors of the PMV model are significantly correlated with the RMOT in a fully air-conditioned museum environment. Correcting the PMV model's prediction by including RMOT improves the explained variance of predicted versus AMVs and brings the linear regression slope closer to the line of identity: slope_PMV_ = 1.58 and slope_PMV*_ = 1.27 (slope = 1 if PMV and AMV are exactly equal). Hence, it has been demonstrated that the prediction accuracy may be improved by extending the PMV model by additionally including the variable RMOT.

Although the new slope of PMV* is closer to the line of identity, it still results in underestimation of the AMV at the progressively cool (AMV < −0.5) and warm (AMV > 0.5) sides of the thermal sensation spectrum. The remaining variance and bias could not be explained further by clothing level as the significant correlations between the residuals (AMV − PMV*) and clothing level (Table 5) were no longer present after inclusion of RMOT in PMV* (Table 6). This is consistent with earlier findings on the relation between outdoor temperature and clothing level as well as other factors that change with RMOT, e.g., behavior, expectation, and acclimatization.^24^

Further analysis revealed that the remaining residuals (AMV − PMV*) strongly correlate with the variable thermal preference. Thermal preference is, in general, not a variable that is known beforehand and is therefore of little practical importance in a predictive model. Nevertheless, including thermal preference has explained the underestimation of the PMV* model toward more extreme AMVs: The new slope is nearly identical to the line of identity (see Fig. 9), but the explained variance has not improved (*R*^2^-value remains 0.8). The reason why thermal preference seems to remove most of the prediction bias may be twofold: (1) thermal preference may be interpreted as being the inverse of thermal sensation, and hence, it may relate strongly to the actual thermal sensation: People that feel cold indicate they prefer a warmer environment,^25^ or (2) visitors expected a neutral environment and were more critical to any deviation toward warmer or cooler environments. The latter is in line with thermal expectation, which has been shown to improve thermal sensation prediction in NV buildings.^7^

In conclusion, the Hermitage Amsterdam museum was used as a case study on thermal sensation of its visitors. The museum is a public space visited by people that vary widely in age and with increasing age comes a decreasing metabolic rate. A metabolic rate is one of the key parameters that is used in the prediction of thermal sensation based on physical heat balance. This study investigated what value for the metabolic rate would best suit the prediction of thermal sensation. Furthermore, the added value of including information on the outdoor temperature (RMOT) has been explored.

The results show that

1. The optimal metabolic rate for thermal sensation prediction using the PMV model was 1.51 Met for a museum environment that was predominantly visited by elderly females. The optimal value is very close to the *a priori* selected value of 1.50 Met; however, this *a priori* selected value may be substantially off in other cases, for example, in museums mostly visited by younger people.
2. Significant differences in metabolic rate were revealed between adults and elderly subpopulations (1.67 Met for adult males vs. 1.50 Met for elderly males, and 1.60 Met for adult females vs. 1.44 Met for elderly females), which are in line with what would be expected based on empirical models that predict metabolic rate. Hence, the results show the importance of differentiating between subpopulations when determining the metabolic rate. The standard tabular values, which only differentiate between various activities, may be oversimplified for many cases.
3. Extending the PMV model with the variable RMOT significantly improved the thermal sensation prediction, but the thermal sensation toward extreme cool and warm sensations remained partly underestimated.

## Abbreviations

AMV: actual mean vote
ASHRAE: American society of heating refrigerating and air-conditioning engineers
PMV: predicted mean vote
PPD: percentage people dissatisfied
RMOT: running mean outdoor temperature
TSV: thermal sensation vote
